# Artificial intelligence assessment of the potential of tocilizumab along with corticosteroids therapy for the management of COVID-19 evoked acute respiratory distress syndrome

**DOI:** 10.1371/journal.pone.0280677

**Published:** 2023-02-15

**Authors:** Cristina Segú-Vergés, Laura Artigas, Mireia Coma, Richard W. Peck

**Affiliations:** 1 Anaxomics Biotech, Barcelona, Spain; 2 Research Programme on Biomedical Informatics (GRIB), Departament de Ciències Experimentals i de la Salut, Universitat Pompeu Fabra, Barcelona, Spain; 3 Pharma Research & Development (pRED), F. Hoffman-La Roche Ltd., Basel, Switzerland; 4 Department of Pharmacology & Therapeutics, University of Liverpool, Liverpool, United Kingdom; University of Kansas Medical Center, UNITED STATES

## Abstract

Acute respiratory distress syndrome (ARDS), associated with high mortality rate, affects up to 67% of hospitalized COVID-19 patients. Early evidence indicated that the pathogenesis of COVID-19 evoked ARDS is, at least partially, mediated by hyperinflammatory cytokine storm in which interleukin 6 (IL-6) plays an essential role. The corticosteroid dexamethasone is an effective treatment for severe COVID-19 related ARDS. However, trials of other immunomodulatory therapies, including anti-IL6 agents such as tocilizumab and sarilumab, have shown limited evidence of benefit as monotherapy. But recently published large trials have reported added benefit of tocilizumab in combination with dexamethasone in severe COVID-19 related ARDS. *In silico* tools can be useful to shed light on the mechanisms evoked by SARS-CoV-2 infection and of the potential therapeutic approaches. Therapeutic performance mapping system (TPMS), based on systems biology and artificial intelligence, integrate available biological, pharmacological and medical knowledge to create mathematical models of the disease. This technology was used to identify the pharmacological mechanism of dexamethasone, with or without tocilizumab, in the management of COVID-19 evoked ARDS. The results showed that while dexamethasone would be addressing a wider range of pathological processes with low intensity, tocilizumab might provide a more direct and intense effect upon the cytokine storm. Based on this *in silico* study, we conclude that the use of tocilizumab alongside dexamethasone is predicted to induce a synergistic effect in dampening inflammation and subsequent pathological processes, supporting the beneficial effect of the combined therapy in critically ill patients. Future research will allow identifying the ideal subpopulation of patients that would benefit better from this combined treatment.

## 1. Introduction

Since the end of 2019 until now, the worldwide coronavirus disease 2019 (COVID-19) pandemic has affected millions of people, caused a global emergency, and had a terrible impact on global public health [1, 2]. COVID-19 has shown an extremely variable clinical course, ranging from asymptomatic forms or mild symptoms, with a favourable prognosis, to pneumonia and hypoxemia. Acute respiratory distress syndrome (ARDS) develops in 20 to 67% of hospitalized COVID-19 patients, 42% of patients with pneumonia, and up to 80% of those requiring intensive care, reaching 100% in mechanically ventilated patients [2–5]. ARDS is characterized by pulmonary oedema, hypoxemia, need for pulmonary ventilation and intensive care unit (ICU) admission, and it is the main cause of death in patients with COVID-19 [2–11].

Early evidence indicated that the pathogenesis of COVID-19 evoked ARDS is, at least partially, mediated by a hyperinflammatory cytokine storm in response to viral infection [7, 8, 12, 13]. Studies’ results suggested the likely benefits of immunomodulatory therapies to abrogate the hyperinflammatory process and prevent ARDS development [7, 14]. Dexamethasone is one of the most commonly used glucocorticoids, with high specificity for the glucocorticoid receptor (GR) and high bioavailability [15]. Since glucocorticoids have well-known anti-inflammatory and immunosuppressive properties, they were proposed early on as possible candidates for dampening hyperinflammation in COVID-19 [15–17]. The RECOVERY trial (NCT04381936) established that moderate doses of dexamethasone reduce mortality in severe COVID-19 patients that required mechanical ventilation or supplemental oxygen therapy [18]. A meta-analysis and three other trials confirmed a reduction in mortality by glucocorticoids [19]. Several recently published trials also accrued additional data pointing out to a strong recommendation to treat patients suffering ARDS with corticosteroids [20]. However, many clinically important questions remain unanswered. Dexamethasone has multiple actions related to anti-inflammatory and immunosuppressive effects [21, 22], some would be expected to be beneficial, whilst other might not. Moreover, it remains to be elucidated how dexamethasone interacts with other therapeutic agents [23].

The hyperinflammatory COVID-19 response is mediated, at least partially by the rapid proliferation and/or activation of immune cells and overproduction of pro-inflammatory cytokines, such as interleukin 6 (IL-6) and the tumour necrosis factor (TNF) [7, 8, 12, 13]. In fact, in severe COVID-19 disease, increased levels of IL-6 have been found to be a key factor associated with inflammation [24] and circulating IL-6 levels are closely linked to the severity of COVID-19 infection, implying a possible cytokine-mediated lung damage pathogenesis [25]. These findings suggested the likely benefits of immunomodulatory therapies to abrogate the hyperinflammatory process and prevent ARDS development in COVID-19 patients [7, 14]. Tocilizumab interferes with the binding of IL-6 to its receptor (IL6RA) in a competitive manner, blocking the initiation of the transmembrane signalling cascade [26] and it has been approved for several inflammatory or autoimmune conditions [27]. Recently, the results of several studies and randomized controlled trials (RCTs) investigating the effect of tocilizumab for COVID-19 patients have been published [1], suggesting that the treatment with tocilizumab may improve outcomes in severe COVID-19 patients [6, 28, 29]. In fact, multiple observational and retrospective studies in medical centres all over the world have reported a positive effect of tocilizumab on mortality reduction in severe and critically ill patients [9, 29–35]. However, preliminary results from RCTs, such as the CORIMUNO-TOCI (NCT04331808) [36], COVACTA (NCT04320615) [37] and EMPACTA (NCT04372186) [38], have only achieved mixed conclusions, since some trials reports benefits and others do not [36–38]. Studies with other IL-6 blockers, such as sarilumab, have not yielded more convincing results either [39]. These inconclusive results have raised some questions regarding the efficacy and the best time in the disease course to use this treatment. However, the results of REMAP-CAP [40] and RECOVERY trials [41], showed that corticosteroid therapy along with tocilizumab is associated with improved clinical outcome compared to corticosteroids alone [42, 43]. Yet, the treatment benefits and mechanisms behind them are still unknown and therapeutic timing may still need to be optimized.

The aim of the present study is to identify the molecular mechanisms of action (MoA) of dexamethasone and tocilizumab treatment, as monotherapy and in combination, and their potential in the management of COVID-19 evoked ARDS. For this purpose, we used an artificial intelligence (AI)-based analysis, which is a powerful tool to deepen the understanding of drugs effects in complex clinical settings and obtain mechanistic insight on efficacy [44]. AI and *in silico* systems biology methods have been applied in the current global pandemic as a mean to investigate therapeutic options in a fast and efficient way [45, 46]. Our analysis offers a means to investigate the specific MoAs of dexamethasone along with tocilizumab in COVID-19 patients.

## 2. Material and methods

**Fig 1:**
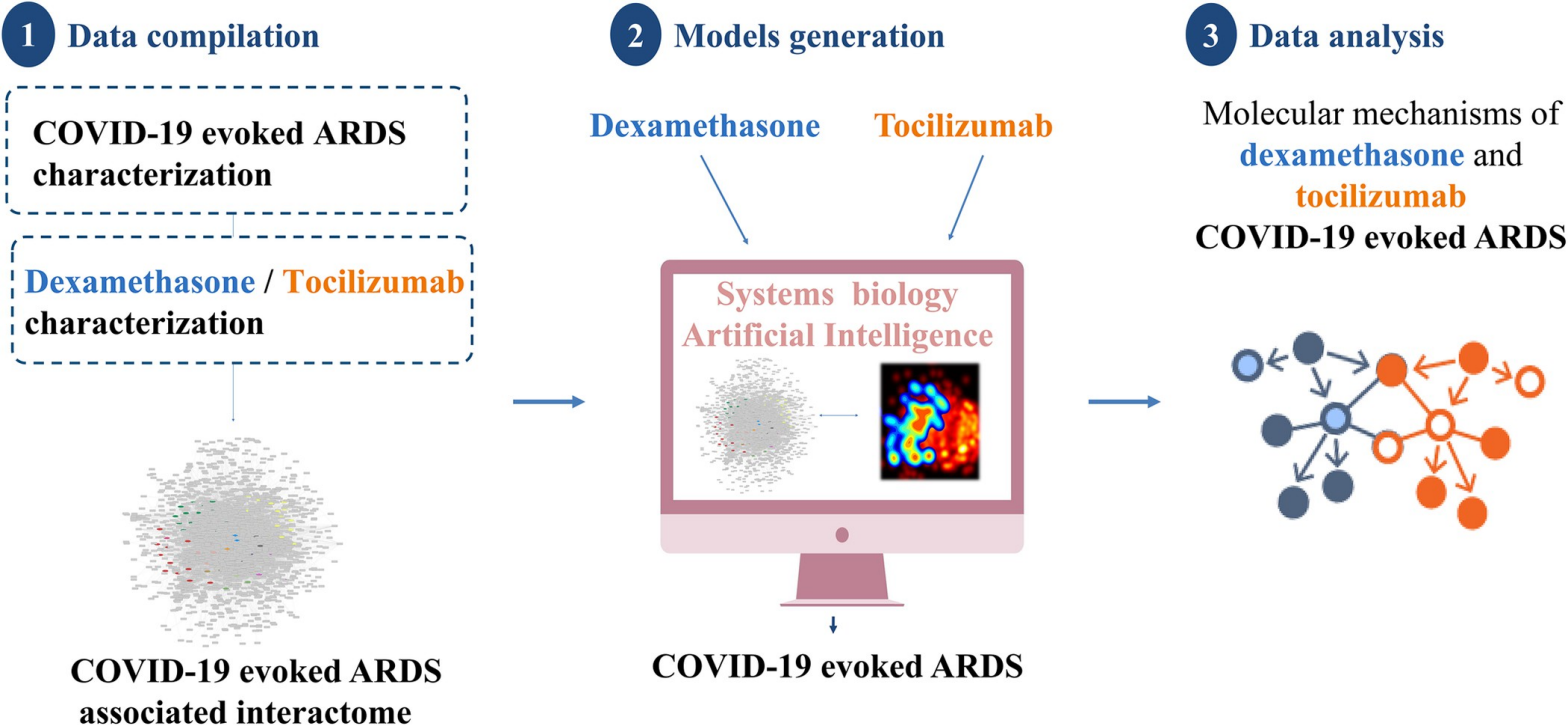
Study workflow. Overview of the *in silico* approach showing the main phases employed to simulate the MoA of dexamethasone and tocilizumab with respect to COVID-19 evoked ARDS, including: 1) data compilation for molecular characterization of COVID-19 evoked ARDS, dexamethasone and tocilizumab obtained from literature review; 2) mathematical model generation using the validated top-down systems biology- and AI-based TPMS approach; and 3) data analyses employing model outputs. AI, artificial intelligence; ARDS, Acute respiratory distress syndrome; MoA, mechanism of action; TPMS, Therapeutic Performing Mapping Systems.

Fig 1 summarizes the workflow and key steps used to identify candidate pharmacological mechanisms of dexamethasone as well as tocilizumab in the management of COVID-19 evoked ARDS by applying machine-learning based technology, the Therapeutic Performing Mapping Systems (TPMS) technology [47].

### 2.1 Molecular characterization of COVID-19 evoked ARDS and drugs under study

The study started by compiling all available COVID-19 and drugs under study available information (Fig 1, step 1). COVID-19 evoked ARDS was molecularly characterized through manual curation of the current scientific literature regarding this subject after a bibliographical search of the available knowledge in PubMed, Google Scholar, bioRxiv, and medRxiv. The following search string was used (all until July 2020): *((wuhan[Title/Abstract] AND ("coronavirus"[Title]) OR "2019nCoV"[Title] OR "COVID-19"[Title] OR "SARS-CoV-2"[Title] OR " SARS coronavirus 2"[Title]) AND ("molecular"[Title] OR "profile"[Title] OR "patients"[Title] OR "expression"[Title] OR "fingerprint"[Title] OR "profile"[Title] OR "high-throughput"[Title] OR "transcriptomics"[Title] OR "proteomics"[Title]) AND ("cytokine storm"[Title/abstract] OR "cytokine storms"[Title/abstract] OR "cytokine release"[Title/abstract] OR "acute respiratory distress"[Title/abstract] OR "acute respiratory syndrome"[Title/abstract]))*. The results retrieved were assessed at the abstract or full-length level, depending on whether they contained molecular information. The main pathophysiological processes (motives) identified to be involved in COVID-19 were: “Initial pro-inflammatory cytokine response”, “Migration of immune cells to pulmonary tissue”, “Adaptive immune system activation”, “Pneumonia and shortness of breath”, “Inflammatory cytokine storm”, “Lung epithelial damage” and “Pulmonary fibrosis”. The last four motives were identified as the key manifestations of COVID-19 evoked ARDS, and they were further characterized at protein level (S1 Table), with a total of 66 non-duplicated protein effectors identified (S2 Table).

The drugs of interest in this project, dexamethasone and tocilizumab, were molecularly characterized through the identification of drug targets (Table 1) and genes or proteins whose activity or expression is modified due to the drug downstream effect, while not being a direct drug target (namely bioflags), (S3 Table) according to a review of official regulatory (EMA and FDA) documentation and scientific databases (DrugBank [48, 49], Stitch [50] and SuperTarget [51]) and literature. All results from the following searches were assessed at the abstract and/or full-length level: *("dexamethasone" [title/abstract] AND ("molecular" OR "mechanism" [title/abstract]) AND ("covid-19" OR "coronavirus" OR "sars-cov-2" OR "2019nCoV" OR "SARS coronavirus 2"[title/abstract]); ("tocilizumab" OR "atlizumab" [title/abstract] AND ("molecular" OR "mechanism" [title/abstract]) AND ("covid-19" OR "coronavirus" OR "sars-cov-2" OR "2019nCoV" OR "SARS coronavirus 2"[title/abstract])*).

**Table 1:** Summary of the drug characterization at target level.

Drug	Protein Name	Gene Name	UniProt ID	Effect	Reference
DXM	Glucocorticoid receptor	NR3C1	P04150	**act**	[52]
DXM	Nuclear receptor subfamily 0 group B member 1	NR0B1	P51843	**act**	[53]
TCZ	Interleuking-6 receptor subunit alpha	IL6RA	P08887	**inh**	[54]

Dexamethasone (DXM) and Tocilizumab (TCZ) targets considered for mechanism modelling. The effect of the drug over the proposed target is indicated by inh (inhibition) or act(activation).

### 2.2 TPMS technology: Systems biology-based model creation

TPMS (Anaxomics Biotech, Barcelona, Spain) is a validated top-down systems biology approach based on AI and pattern recognition models that integrates all available biological, pharmacological, and medical knowledge, to generate models that simulate *in silico* the behaviour of human physiology (Fig 1, step 2) [47, 55]. TPMS starts of the whole human protein-protein interaction network. The protein-protein interaction human network incorporates the available relationships (edges or links) between proteins (nodes) from a regularly updated in-house database drawn from public sources, as previously described [47, 55]. In order to transform this network or map into mathematical models capable of both reproducing existing knowledge and predicting new data, a collection of known input-output physiological relationships is used as train data, or ‘truth table’, to train the models. The latter is constructed using a compendium of biological and clinical databases through text mining techniques and manual review and curation of the information to obtain biological and pharmacological input-output relationships (such as drug-indication pairs) [47–49]. Finally, for a given study’s input (eg. drug targets) and output (eg. pathology characterized proteins), a sampling-based approach is applied to generate models similar to a Multilayer Perceptron of an Artificial Neural Network over the human protein network (where neurons are the proteins, and the map edges/links are used to transfer the activation/inhibition signal information). To do so, each link in the network is assigned a weight and the signal is propagated across the system starting from input. In order to make the model behave according to human physiology, truth table restrictions are applied a training data. Since the number of entries in the truth table is always smaller than the number of parameters (link weights) required by the TPMS algorithm, the methodology considers a population of different solutions. Focus is then set to the different paths of proteins and links between input and output protein sets, which will form with varying probability, and create a universe of diverse plausible solutions. The resulting model, consistinting on a set of solutions, is able to describe all possible mechanistic relationships, or MoAs, between input and output. Each solution accuracy is calculated as the percentage of all the truth table rules with which it does comply, while accuracy of the final model was calculated as the mean of the accuracies of all considered solutions.

Specific COVID-19-evoked ARDS networks and mechanistic models are constructed using as starting material the list of proteins effectors and targets obtained from literature review that were observed in tissue and/or blood of COVID-19 evoked ARDS patients (S2 Table, Table 1). For visualizing the molecular interaction of COVID-19 evoked ARDS network we used the Cytoscape software v.3.7.0 (https://cytoscape.org/) [56].

To obtain the COVID19 evoked ARDS mathematical models, the human protein-protein interaction network was trained using artificial intelligence approaches, as described above [47], to comply with a set of known human physiological rules, namely relationships between drugs, defined as their protein targets, and clinical conditions and indications, defined as proteins involved in the condition (TPMS training set, S4 Table). These models were constructed using drug targets (Table 1) as stimulus and “Inflammatory cytokine storm”, “Lung epithelial damage”, and “Pulmonary fibrosis” (S2 Table) motives as response. No patient data were included as models constrains. Given that COVID-19 evoked ARDS patients already display pneumonia and shortness of breath signs in the moment of diagnosis, we included “Pneumonia and shortness of breath” motive (S2 Table) as basal clinical patient situation. Furthermore, known pharmacological effects of each drug (drug bioflags) were also included as model constrains for more accurate MoA modelling.

At this point, each link in the protein network is assigned a weight to propagate the signal from drug targets to proteins involved in diseases. Paths of proteins and links between them will form with varying probability, in order to allow the model to behave according to human physiology, and create a universe of diverse plausible solutions, both from a probabilistic and biological point of view [47, 55], and then to obtain all MoA solutions of the effects of dexamethasone and tocilizumab over ARDS molecular players. Only solutions with accuracy higher than 90% (percentage of compliance of all drug-clinical condition relationships included in the training set) were considered as valid. Accuracy of the final MoA models was calculated as the mean of the accuracies of all considered solutions. Once identified the individual mechanisms for each drug, they were jointly evaluated to identify the potential of the combination on the management of COVID-19 evoked ARDS.

### 2.3 Measures and data analyses

Predicted protein activity (in a range from 1 to -1) was obtained from the TPMS models [47, 55, 57] for data analysis (Fig 1, step 3). This value for the ARDS effectors was analysed per protein individually and combined to obtain an aggregated molecular measure, or “intensity” of the response. COVID-19 evoked ARDS protein effectors were considered as *reversed* towards recovery by the drugs when their predicted protein activity was of the opposite sign to the one defined in the disease molecular characterization (i.e., activated proteins that are inhibited in the disease, and vice versa) and displayed a |predicted protein activity| ≥ 0.1. ARDS effectors were used to calculate the intensity which is defined by three measures: 1) the number of protein effectors (#Eff) achieving the expected signal to ameliorate a disease; 2) a measure of the strength of the output signal of the effectors (i.e., a global measure of the output signal, named T-Signal [47, 55, 57]); and 3) a variation of this measure that weights the output signal of effectors on the whole pathology definition (i.e., a weighted global measure of the output signal which ponders both the number of effectors correctly (#*Eff*) and incorrectly ($\overset{-}{\#}Eff)$ activated and the amount of signal that they receive, named W-Signal, Eq 1).


$$WSignal\;=\;\left(1+\frac{\#Eff+1}{\overset{-}{\#}Eff+1}\right)\;*\left(\frac{\#Eff}{{BED}_{p}}+TSignal\right)$$ Equation 1

were BED_p_ is the total number of effectors for each definition evaluated.

The proteins upregulated (predicted protein activity ≥ 0.1) and downregulated (predicted protein activity ≤ 0.1) by each drug (dexamethasone and tocilizumab) in the mathematical models were submitted to independent hypergeometric enrichment analyses [58], to identify common and differential pathways and molecular processes modulated by dexamethasone and tocilizumab in the COVID-19 evoked ARDS patients. Specifically, the enrichment was run over the following databases: GO (Gene Ontology) terms (Biological Process and Cellular Component) [59, 60], KEGG (Kyoto Encyclopedia of Genes and Genomes) pathways [61, 62], and pathological conditions and motives included in the BED (Biological Effectors Database, Anaxomics property databases) [47, 63]. A significance threshold has been set for the analysis in a false discovery rate (FDR, Benjamini-Hochberg multi-test) p-value < 0.05, while those pathways containing 500 or more genes in their description have been excluded, with the aim of filtering unspecific results.

## 3. Results

### 3.1 COVID-19 evoked ARDS associated interactome

**Fig 2:**
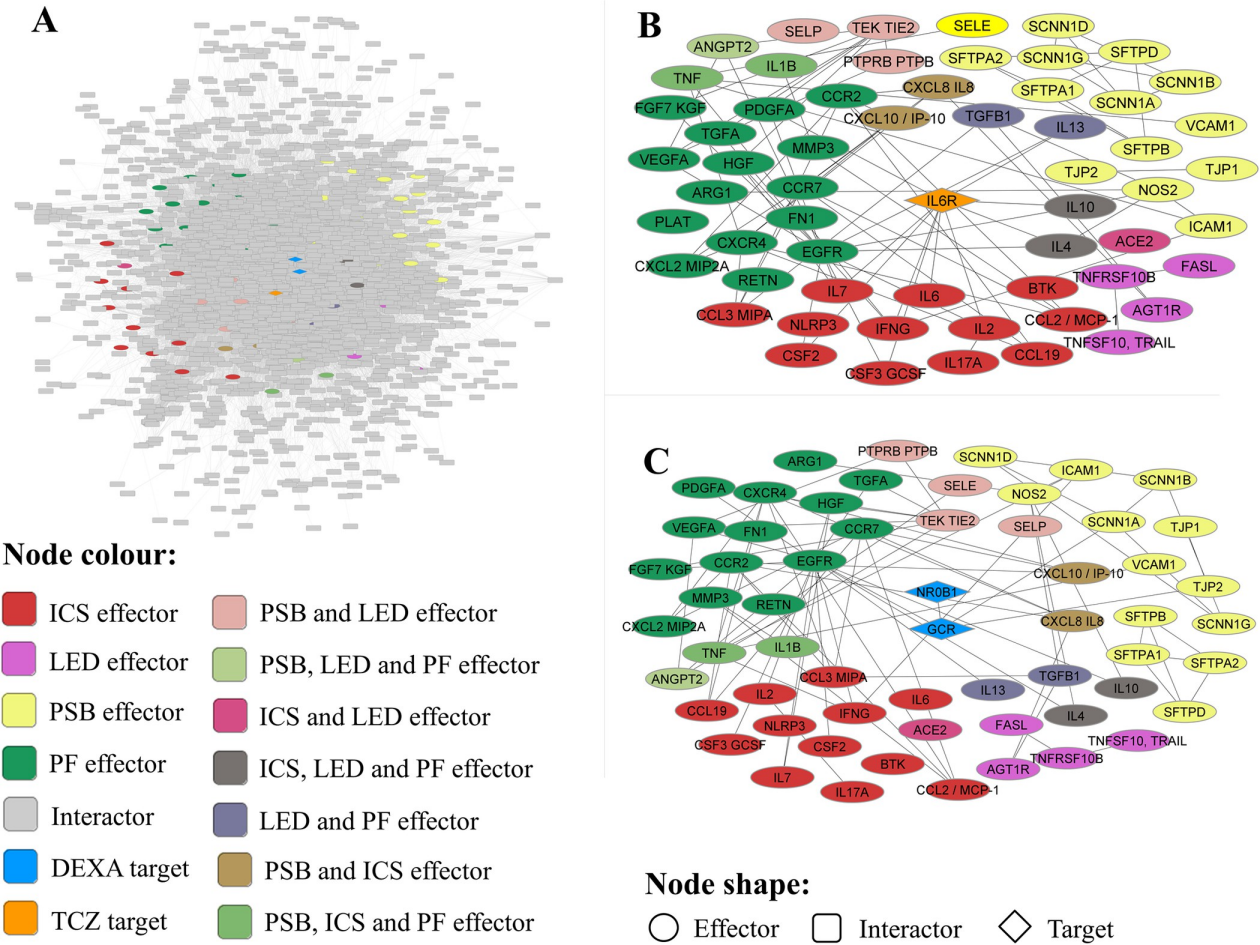
COVID-19 evoked ARDS interactome. Human protein networks around COVID-19 evoked ARDS molecular pathophysiology considering all disease effectors and their direct interactors. General overview (A) and centred on the relationship of COVID-19 evoked ARDS effectors to tocilizumab (B) and dexamethasone (C) drug targets. Image created with Cytoscape 3.7.0 [56]. DEXA, dexamethasone; ICS, Inflammatory cytokine storm; LED, Lung epithelial damage; PF, Pulmonary fibrosis; PSB, Pneumonia and shortness of breath; TCZ, tocilizumab.

A key element of model creation is to build a protein-protein interaction network based around the identified COVID-19 evoked ARDS effectors (see S1 and S2 Tables), which serves to place these identified effectors within the broader context of all reported human protein-protein interactions (S1 File; Fig 2A). The complex COVID-19 evoked ARDS interactome includes the protein targets from both drugs (Fig 2B and 2C), which interact with a comparable number of COVID-19 evoked ARDS effectors, assigned to the four motives: 1) “Pneumonia and shortness of breath”, (2) “Inflammatory cytokine storm”, (3) “Lung epithelial damage”, and (4) “Pulmonary fibrosis”.

### 3.2 Tocilizumab and dexamethasone mechanisms on COVID-19 evoked ARDS

**Fig 3:**
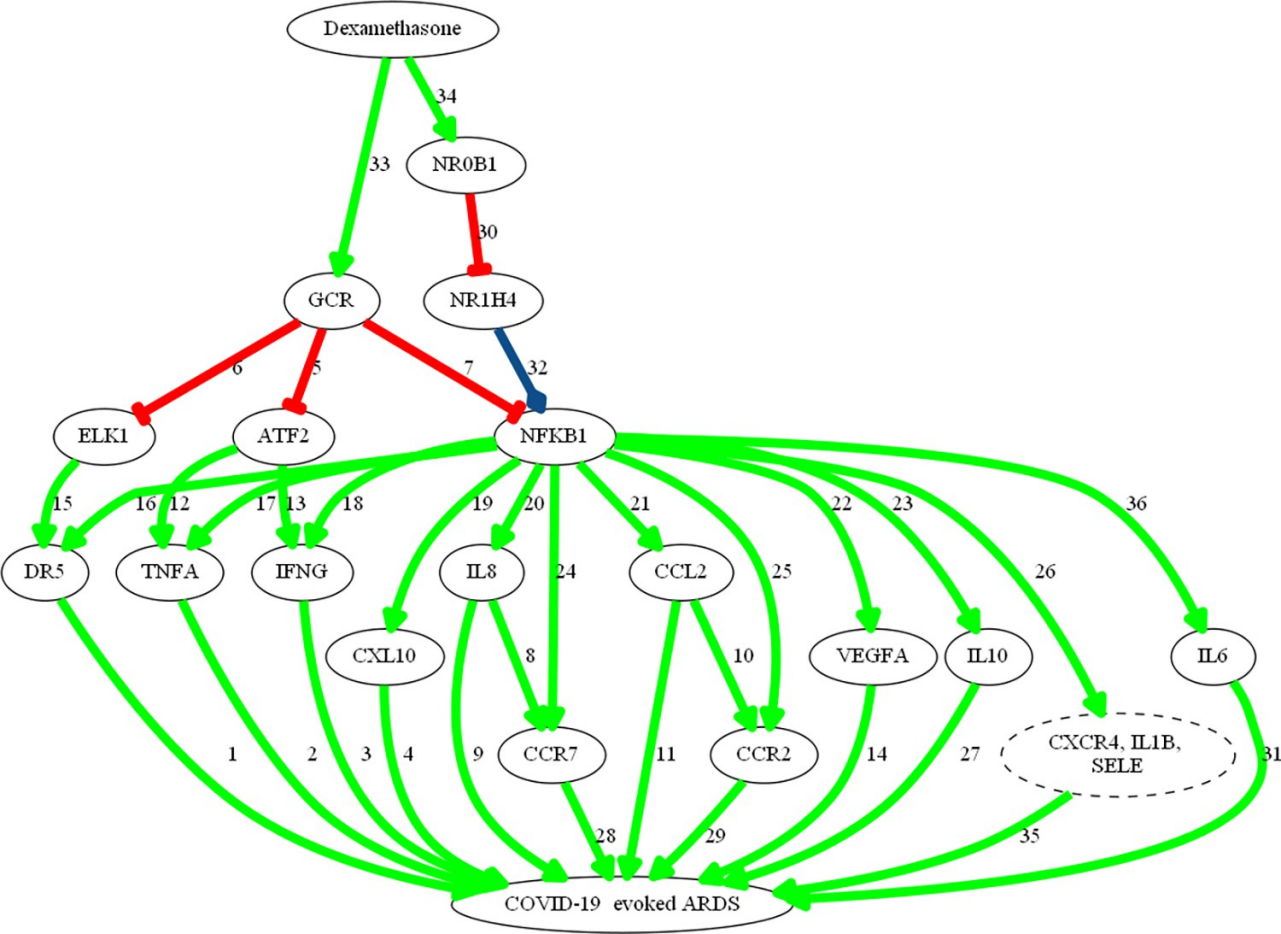
Mechanism of action of dexamethasone. Figure created to represent TPMS MoA predictions using Graphviz software. All links have been manually reviewed: the number of the links correspond to the reference code (link number) in S5 Table. Green arrows show activation; red line shows inhibition; blue lines show complex or dual relationships; broken-lined circles indicate a node that contains more than one protein, all acting in the MoA in the same way.

**Fig 4:**
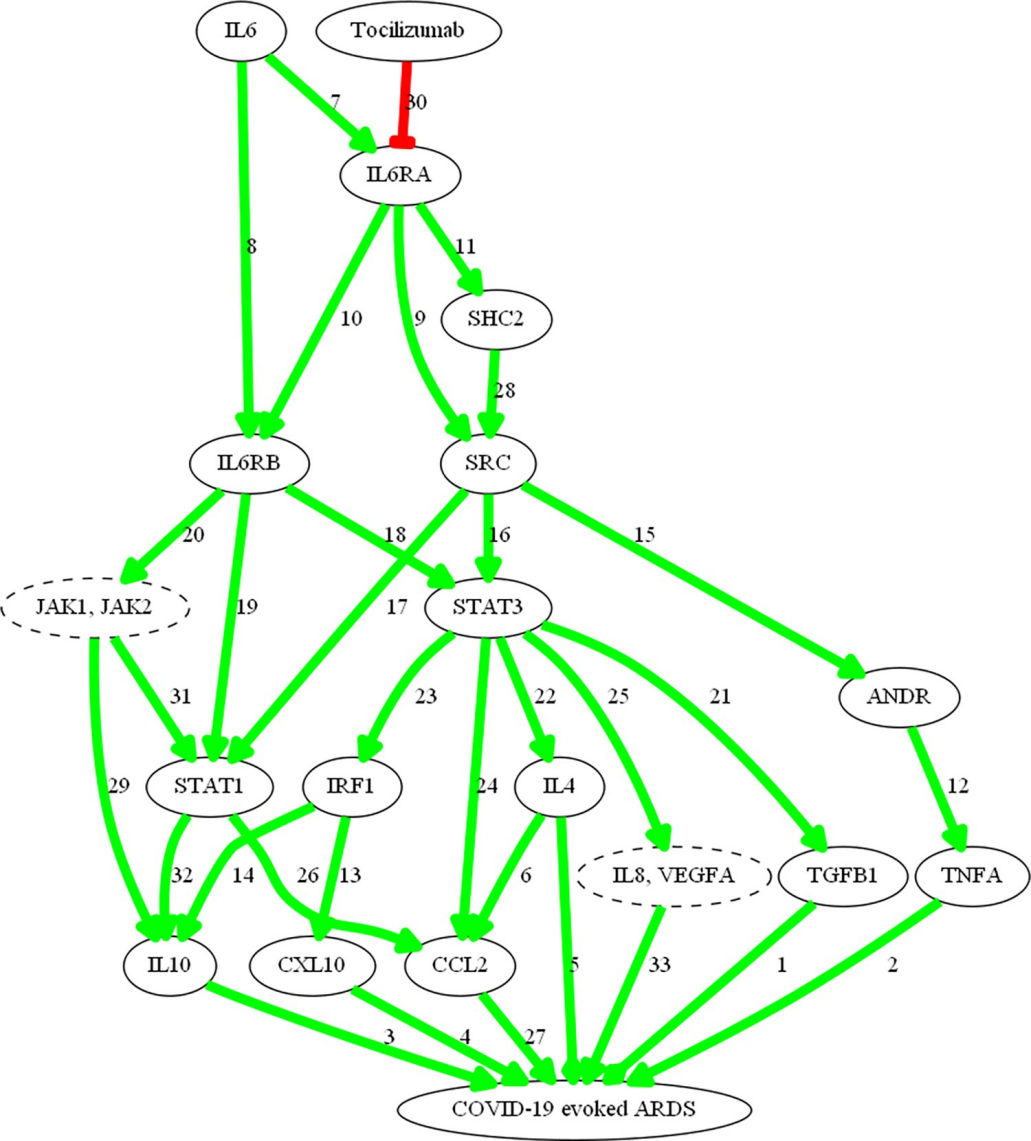
Mechanism of action of tocilizumab. Figure created to represent TPMS MoA predictions using Graphviz software. All links have been manually reviewed: the number of the links correspond to the reference code (link number) in S6 Table. Green arrows show activation; red line shows inhibition; broken-lined circles indicate a node that contains more than one protein, all acting in the MoA in the same way.

The mechanisms of tocilizumab and dexamethasone over ARDS pathophysiological motives used as model response (“Inflammatory cytokine storm”, “Lung epithelial damage” and “Pulmonary fibrosis”) were calculated and a set of 250 biologically plausible solutions were obtained, with a mean accuracy against the training set of a 94%. The most probable (both from a probabilistic and biological point of view) MoA of each drug to treat COVID-19 evoked ARDS (Figs 3 and 4) were elucidated.

Dexamethasone, by targeting the glucocorticoid receptor (Fig 3) is able to modulate COVID-19-evoked ARDS through pathways with wide effects on inflammatory factors, such as NF-κB, IL-1β, TNFα and IL-6, as well as fibrosis. GCR and NF-κB crosstalk can modulate several proinflammatory and immune processes, from cell recruitment to cytokine production. TNFRSF10B (or TR10B) and TGFB1 regulate lung oedema and fibrosis.

Tocilizumab models (Fig 4) show that, through IL-6 signalling blockage, the drug can alter the activity of several proteins involved in COVID-19-evoked ARDS. Tocilizumab is able to dampen excessive inflammation by two different pathways: (1) limiting excessive immune cell recruitment to the local lung tissue by targeting CCL2/MCP-1, CCL2, CXCL10 and IL-8; and (2) preventing T-cell exhaustion and fibrosis induction by modulation of IL-10, IL-4, CCL2 and TGFB1 levels. Tocilizumab also could reduce the cytokine storm and the subsequent development of oedema and pulmonary fibrosis progression by decreasing VEGFA, IL-4 and especially TFGB1.

### 3.3 Combination mechanisms of tocilizumab plus dexamethasone in the management of COVID-19 evoked ARDS

**Fig 5:**
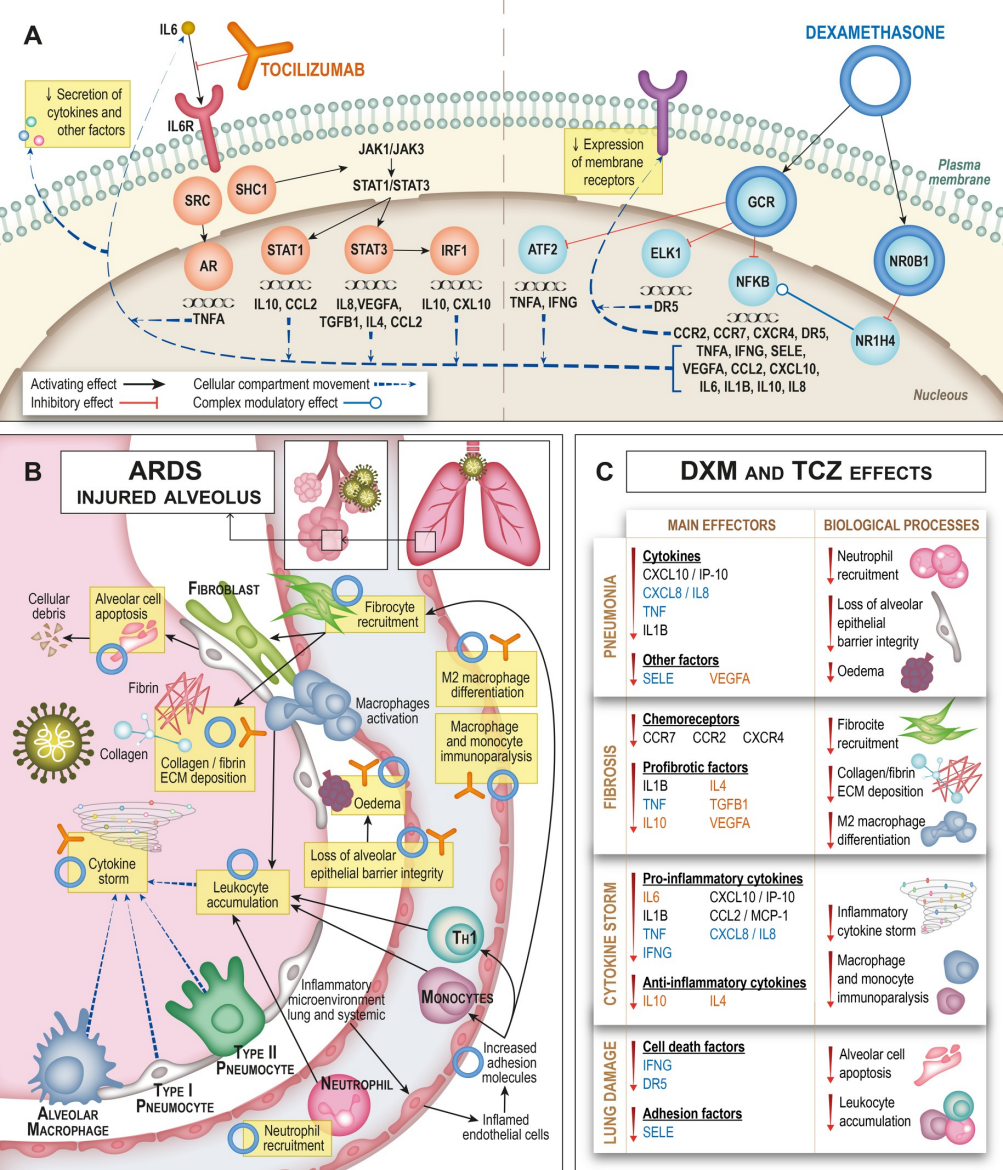
Mechanism of action of dexamethasone and tocilizumab as combined therapy. Consequences of the molecular modulation based on known proteins function and role in COVID-19 evoked ARDS is indicated for biological interpretation. A) shows the intracellular mechanisms of each drug, as predicted by the models; B) shows the known pathological situation in the alveolus in COVID-19 evoked ARDS, immune components implicated and pathological processes, as well as the drugs can action; C) summarises dexamethasone and tocilizumab effects on specific proteins and pathological processes in COVID-19 evoked ARDS; and classified in each of the studied motives (blue: mainly modulated by dexamethasone, orange: mainly modulated by tocilizumab, black: modulated by both drugs).

The predicted MoA for each drug were jointly evaluated, aiming at the elucidation of the likely pharmacological mechanisms evoked by the combination against COVID-19 evoked ARDS (Fig 5).

**Fig 6:**
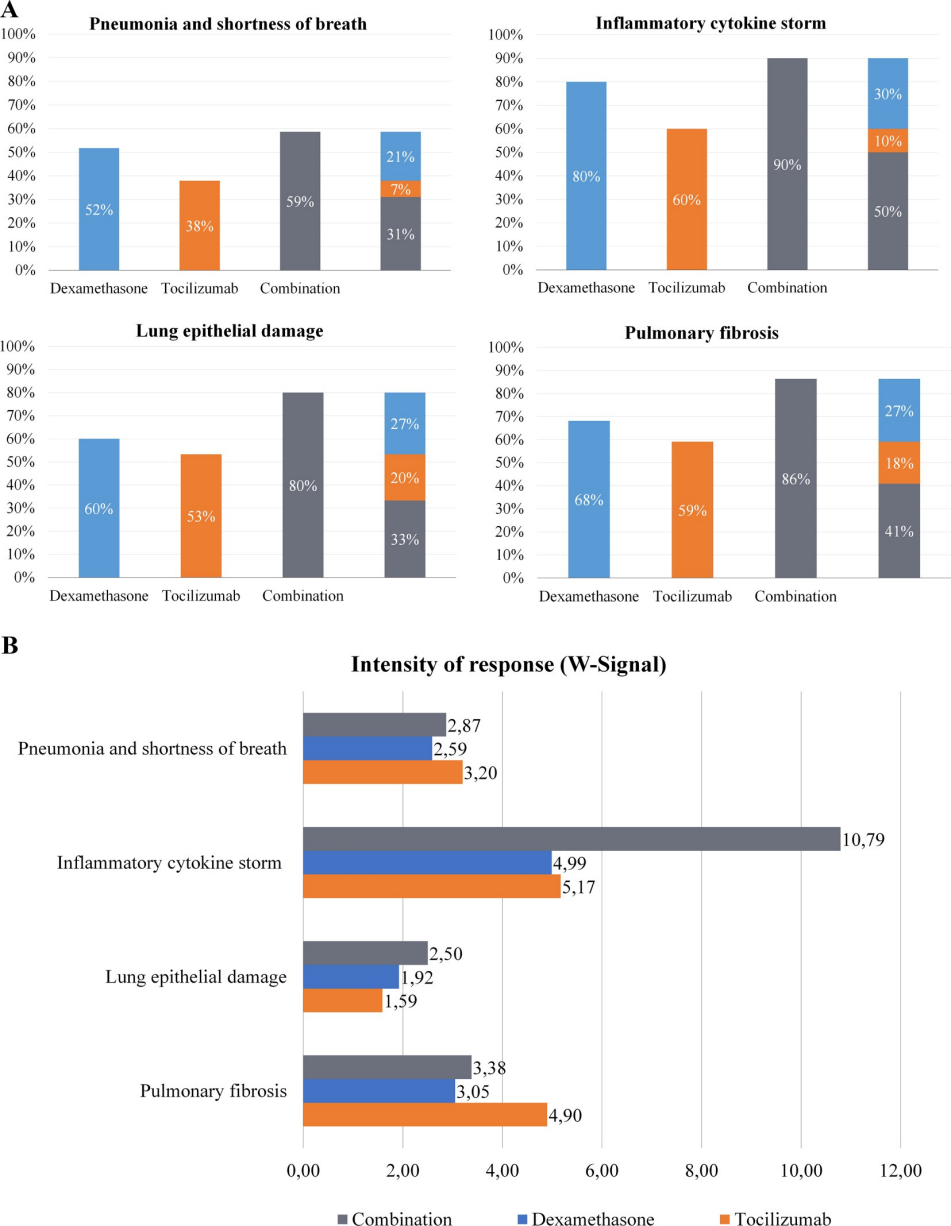
Impact of dexamethasone and tocilizumab over COVID-19 evoked ARDS pathophysiological motives. (A) Percentage of altered effectors reversed by each drug in each specific COVID-19 evoked ARDS motive. (B) Intensity of response (W-Signal) to each drug and the combination in the COVID-19 evoked ARDS specific motive, according to the simulated models.

The impact of each treatment on the specific biological processes (motives) involved in this disease were assessed, through two complementary parameters: (1) Percentage (%) of altered effectors reversed by the drug, and (2) intensity of response defined as W-Signal, a measure that weighs the strength of the output signal of effectors across the whole motive definition, considering the number of effectors reversed and the intensity of such modulation. These parameters provide an idea of the scope and the strength of the impact of the treatment effect on a specific biological process. The combination of tocilizumab and dexamethasone had an impact in the four motives evaluated (Fig 6), but specially in “Inflammatory cytokine storm” and “Pulmonary fibrosis”.

Both drugs were able to revert proteins within the four motives, achieving more than 80% of effectors reversed in “Inflammatory cytokine storm”, “Lung epithelial damage” and “Pulmonary fibrosis”, especially by the impact of dexamethasone (Fig 6A). The evaluation of the W-Signal (Fig 6B) showed that the combination of tocilizumab and dexamethasone had a strong synergistic impact on “Cytokine storm”, while both presented a similar impact when considered individually. When evaluated together with % of effectors reversed (Fig 6A), it was evidenced that, while tocilizumab impacts fewer proteins than dexamethasone, it does it more strongly. Dexamethasone provided a wider coverage in reverting “Pulmonary fibrosis” effectors (Fig 6A), and tocilizumab had a stronger impact individually than dexamethasone and the combination, as measured by the W-Signal (Fig 6B), reflecting a targeted and strong effect over limited fibrosis effectors. Therefore, the concomitant administration of tocilizumab and dexamethasone was predicted to achieve a strong immunomodulatory effect that may attenuate hyperinflammation in COVID-19 evoked ARDS.

**Fig 7:**
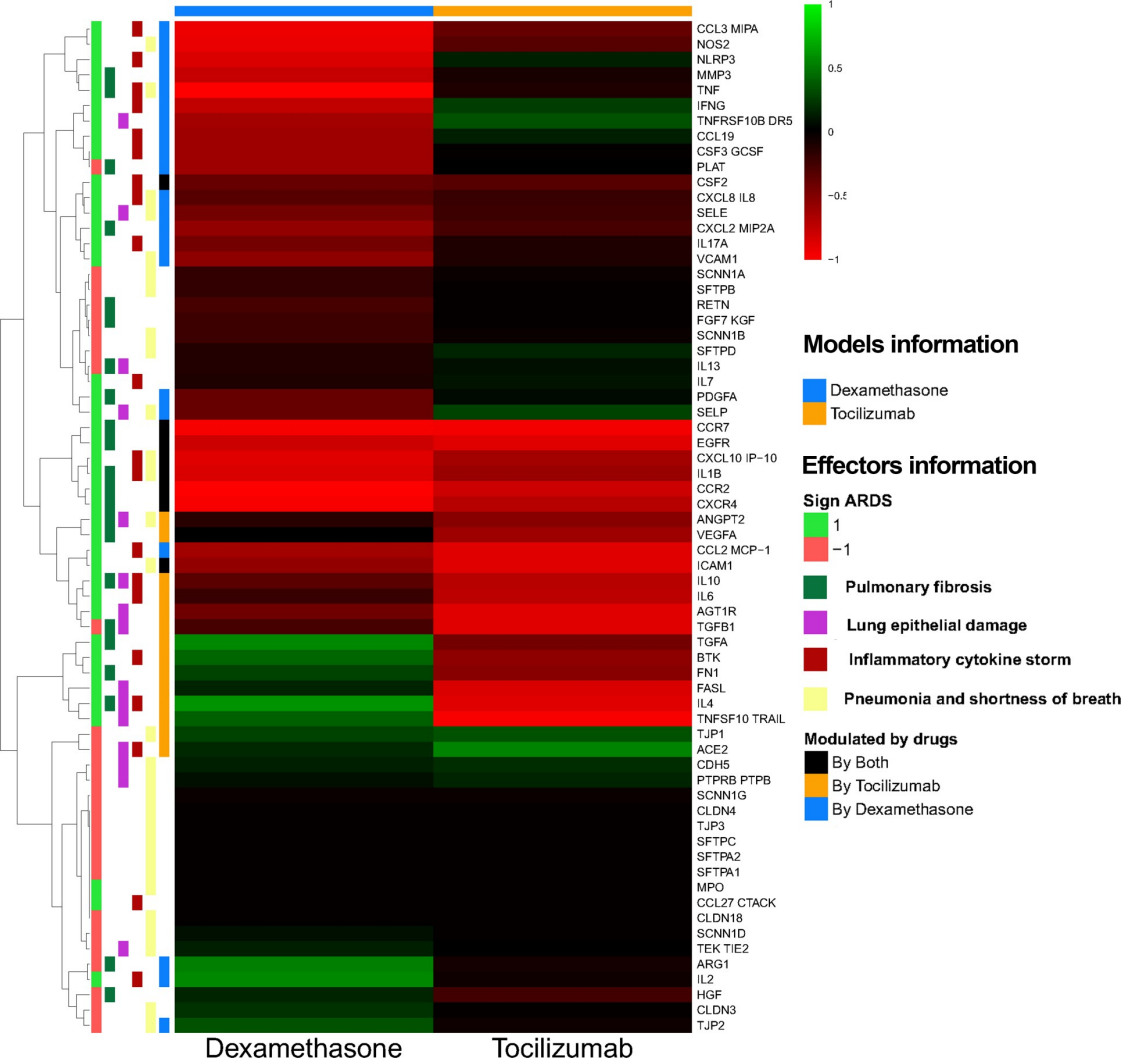
Effector protein heatmap activated for each model (dexamethasone and tocilizumab). Heatmap of the average predicted protein activity induced by dexamethasone and tocilizumab over the effectors of the pathology. The vertical bars indicate the pathological effect of the effectors (*ARDS sign*: 1 if activated in the pathology, -1 if inhibited in the pathology); which COVID-19 evoked ARDS motives the proteins are assigned (*Effector in motive*) and whether the models reflect an effect of each drug over each effector (*Modulated by drugs*, considering |0.3| as the threshold of modulation and of modulation difference between drugs).

To obtain further insight, the mean predicted protein activity of all COVID-19 evoked ARDS effectors (Fig 7) was compared between both drugs. In line with the overall percentage of effectors, dexamethasone also induced stronger reversion of individual proteins, such as chemokines and chemokine receptors (CCL19, CCL3, CCR2, CXCL10, CXCL2 and CXCR4), interleukins (IFNγ, IL-17A, IL-1 β, IL-7 and IL-8), and pulmonary damage (E-selectin–SELE–and P-selectin–SELP) and fibrosis effectors (MMP3, PDGFA, TNF and DR5). However, tocilizumab was able to more strongly reverse a handful of relevant ARDS effectors: 1) obviously, pleiotropic interleukin IL-6 signalling and activity, involved in the cytokine storm induction; 2) BTK, a central signalling molecule in several immune-related processes; and 3) several effectors involved in epithelial and endothelial biology that have been related to epithelial damage and fibrosis, including cytokines (IL-4, IL-10, TGFA, VEGFA), proteins that regulate endothelial function (ACE2, AGT1R, ANGPT2, CDH5, EGFR, FN1, PTPRB, VEGFA) and involved in apoptosis (FASL, TRAIL).

### 3.4 Biological processes modulated by dexamethasone and tocilizumab

**Fig 8:**
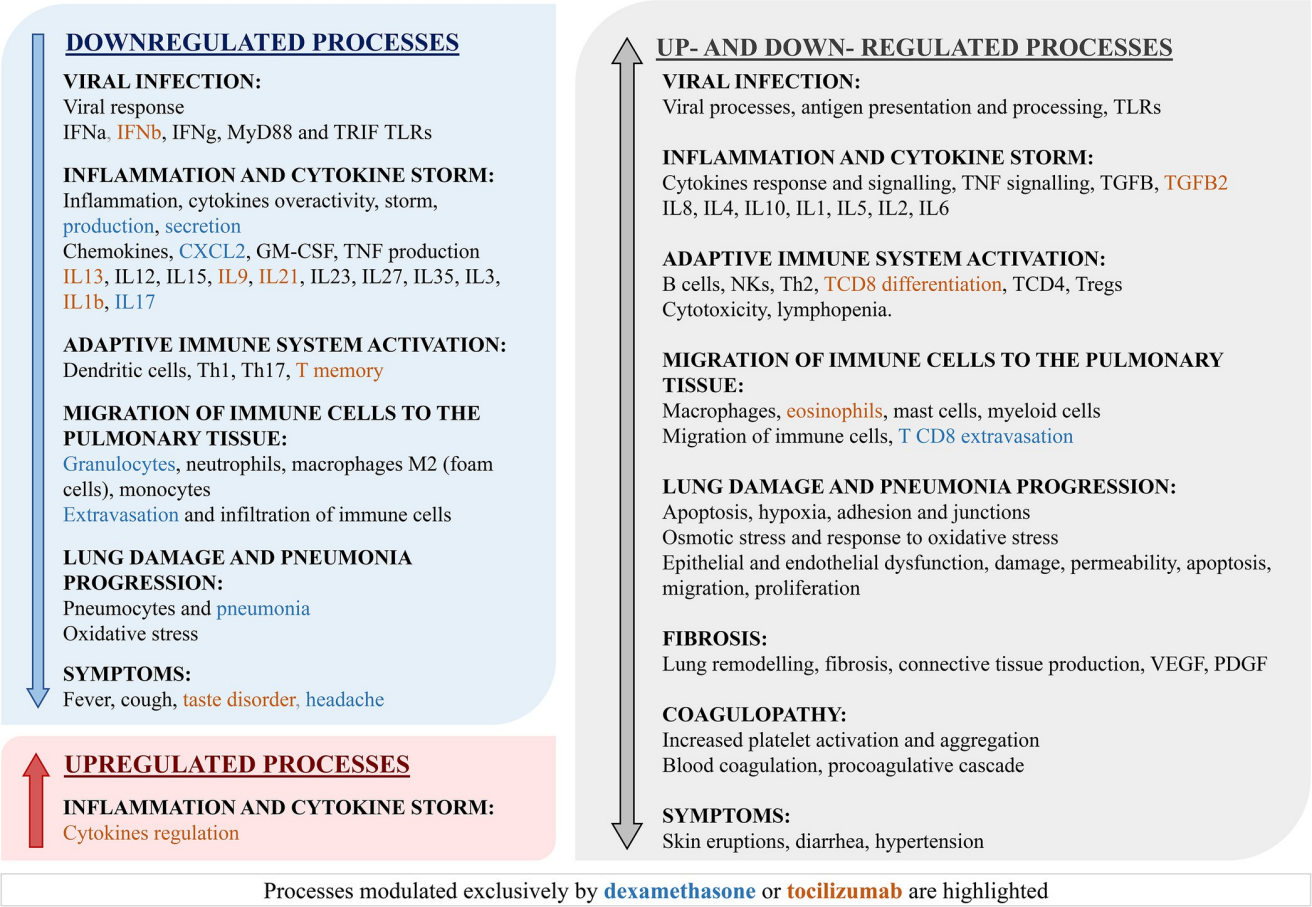
Summary enrichment results. Summary and overview of the biological processes modulated by the combination of dexamethasone and tocilizumab, according to our mathematical models. Proteins activated and inhibited by the pharmacological action of each drug were subjected to hypergeometric enrichment analysis independently, providing upregulated and downregulated processes respectively (modulated processes appear in both upregulated and downregulated analyses). This summary shows the COVID-19 evoked ARDS related processes modulated by the drugs. As indicated in the legend, colours indicate if the effect was shared between drugs (black) or are exclusive of dexamethasone (blue) or tocilizumab (orange).

In order to further contextualize the predicted MoA of the drug combination, the biological processes modulated by these treatments were assessed through hypergeometric enrichment analyses. The processes obtained in the analyses were contextualized in terms of ARDS pathophysiology and classified into categories accordingly (S7 Table), and those ARDS pathophysiology-related processes were evaluated (Fig 8). We separated the proteins upregulated and downregulated by each drug for the analysis, in order to obtain further detail; however, it has to be considered that the biological processes definition provided by the used databases do not necessarily define if the proteins are positively or negatively associated with a process with the exception of the Gene Ontology terms within the “positive regulation” or “negative regulation” categories, (S7 Table). For this reason, we have generally assumed that most of the annotations might reflect a positive association, thus we assigned upregulation to a process when it appeared enriched within the list of proteins upregulated by a drug, and the same for downregulation. This assumption equally affects both mechanisms. While several processes were found downregulated, few were found upregulated; interestingly, the drugs induced both upregulation and downregulation in a number of processes (up- and down- regulated processes).

The enrichment analysis showed effects of both drugs in several immune system mechanisms. Tocilizumab was mainly involved in dampening inflammation, through modulation of interleukin and cytokine regulation, as well as innate and adaptive immune mechanisms and cellular effectors. Dexamethasone results suggested a modulation of a wider range of mechanisms, mainly concerning migration and activity of immune cell types. Interestingly, a potential effect in coagulopathy processes was noted. Dexamethasone provides a clearer downregulation of fibrosis and pneumonia progression related progresses, but some hypercoagulative processes appeared upregulated by this corticosteroid. Moreover, hypertensive processes did not seem clearly downregulated, which could be of concern for specific patient profiles. The results also showed regulation of specific immune response to viruses and effects on other COVID-19 ARDS-related complications, including lung damage and pneumonia progression, coagulopathy, fibrosis, and others.

## 4. Discussion

The results in our study showed the potential molecular mechanism of dexamethasone and tocilizumab for the management of COVID-19 evoked ARDS. Dexamethasone modulated a wider range of protein effectors than tocilizumab, which is mainly involved in dampening the cytokine storm. Tocilizumab might be most useful to treat patients in a disease state marked by an increased inflammatory and immune cellular activity, which is just starting to lead towards tissue damage, critical lung function loss and coagulopathy, such as that reported for patients entering the most critical stage of the disease [2, 6, 8, 10, 11]. Nevertheless, the existence of complementary mechanisms supports the clinical trials evidence that concomitant treatment of severely ill patients with both drugs is more effective than use of either dexamethasone or tocilizumab alone.

Regarding tocilizumab, the STOP-COVID multicentre cohort study [33], and a single centre experience in Spain [30] concluded that the early use of tocilizumab increased patient survival. Despite these positive results, other RCTs, such as the CORIMUNO-TOCI (NCT04331808) [36], COVACTA (NCT04320615) [37] and EMPACTA (NCT04372186) [38], have only achieved mixed or negative conclusions regarding the clinical efficacy of tocilizumab in COVID-19 patients [36–38]. However, these trials did not specifically focus on the most critically ill patients and treatment was not always provided within the first 24h in the ICU. Ongoing clinical trials and preliminary reports on COVID-19 patients treated with sarilumab, another IL-6 blocker, have reported similar inconclusive results [40, 43, 64]. In an open-label cohort study, overall clinical improvement and mortality in patients with severe COVID-19 were not significantly different between sarilumab treatment and standard of care, but there was a faster recovery in a less severe subset of patients [65]. The apparent lack of clear efficacy of IL-6 blocking on COVID-19 can be due to differences in patients’ subpopulations or to differences in trial design. Nevertheless, data so far points out that anti-IL6RA monoclonal antibodies, used as monotherapy, have no significant effect on severe COVID-19 cases, but might have some benefit in less severe patients [40].

The synergistic effect of dexamethasone and tocilizumab is especially relevant in regulating inflammation, where the main collaborative mechanisms are detected, which can lead to modulation of subsequent pathophysiological processes. In this sense, results of REMAP-CAP [40] and RECOVERY [41] trials pointed that corticosteroid therapy along with tocilizumab is associated with improved clinical outcome of patients with COVID-19. Tocilizumab appears to enhance the beneficial effects of dexamethasone in the four COVID-19 evoked ARDS motives, specially by reinforcing the dampening of the cytokine storm and regulating pulmonary fibrosis. Consequently, the concomitant administration of both drugs achieves a strong immune-modulatory effect that may attenuate hyperinflammation in ARDS. Although the current data indicates clinical improvement in adding tocilizumab to dexamethasone in critically ill patients [40, 41], the evidence regarding the role for tocilizumab in regulating inflammation in these patients is scarce. A recent study in critically ill patients treated with dexamethasone show that inflammatory markers, such as the C-reactive protein (CRP), lactate dehydrogenase, ferritin and the neutrophil-lymphocyte ratio were reduced upon tocilizumab treatment (in patients treated with a treatment combination including corticosteroids) [66]. In another study comparing the change from baseline between adding tocilizumab or not, although they found a greater reduction in CRP and ferritin levels when adding tocilizumab, the reduction was not significantly different than the reduction induced by the treatment without tocilizumab [67]; they did find, however, a significant difference when evaluating coagulation parameters, such as activated partial thromboplastin time, prothrombin time and platelet levels. Interestingly, Ponthieux et al. [68], although noting reduced CRP levels, found an increase in IL-1β, -2, -4, -10, -12p70, -18, and sIL-6R days 2–4 after tocilizumab alone treatment in a small cohort of 15 critically ill patients, supporting the relevance of the combination with corticosteroids for an additive anti-inflammatory effect. These evidences point towards an improvement in inflammatory parameters by adding tocilizumab and dexamethasone in critically ill patients.

More specifically, and according to our models, dexamethasone is able to modulate COVID-19 evoked ARDS through pathways with wide effects in inflammation, such as those activated by NF-κB, IL-1β and TNFα [69]. Dexamethasone is a specific agonist of the GR on the cell membrane, forming a complex that leads to translocation of the GR into the cell, where it travels to the nucleus [70]. Glucocorticoid receptor and NF-κB crosstalk activated by dexamethasone, among other factors, modulates several proinflammatory and immune processes, from cell recruitment to cytokine production [71–75]. Tocilizumab interferes with the binding of IL-6 to its receptor (IL6RA) in a competitive manner [26]. IL-6 is one of the most important pro-inflammatory cytokines, with pleiotropic roles in immunity, tissue regeneration, and metabolism. It can be synthesized and secreted by many cell types including monocytes, T-cells, fibroblasts, and endothelial cells [76, 77]. Therefore, tocilizumab can dampen excessive inflammation by limiting excessive immune cell recruitment to the local lung tissue inhibiting the signalling pathways activated by CCL2 [78], CXCL10 [79], IL-8 [80], and TNF [81, 82]. In summary, IL-6 plays a pivotal role in the establishment of a hyperinflammatory milieu that leads to ARDS in patients with severe COVID-19, since it controls the transcription of various cytokines and growth factors involved in inflammation and fibrosis. Hence, a focal point of cooperation between tocilizumab and dexamethasone is the IL-6/IL6-R axis, which is directly targeted by tocilizumab, but also indirectly by dexamethasone.

As detailed, inhibition of IL-6 by tocilizumab may dampen excessive inflammation in initial states of severe disease, avoiding subsequent oedema and fibrosis progression [26, 76, 77], which is reflected in our AI-based analysis through normalization of the inflammatory cytokine storm and pulmonary fibrosis, displaying a high intensity and a moderate coverage. COVID-19 evoked ARDS is part of a complex inflammatory unbalance involving many components of the immune system. This can explain why targeted drugs such as tocilizumab or sarilumab, when used as monotherapy, have shown inconclusive results. Nevertheless, while a broad-spectrum anti-inflammatory drug such as dexamethasone has more extensive and varied effect on inflammation, which is supported by its compelling clinical efficacy, its combination with the more specific and stronger effects of tocilizumab could reinforce and further enhance the effects of dexamethasone. In that sense, targeting several points at the same time might alter feedback loops in the inflammatory cascade and be involved in the proposed synergism between the drugs. Particularly, IL-6 expression is partially under control of NF-κB, which is involved in the signalling of IL-1β or TNFα, and even of IL-6 as secondary mechanism besides the JAK/STAT cascade; in fact, our system models that dexamethasone would regulate the expression of several of these cytokines through this mechanism. Our study’s results, in agreement with published reports, points out to a beneficial combination of both drugs in dampening the cytokine storm by enhancing the effects upon the IL-6 axis. The challenge now remains to identify the population of patients that will benefit most from the combined treatment with dexamethasone and tocilizumab.

Similar studies performed in other clinical conditions demonstrate the potential clinical-translational use of TPMS [83, 84]. However, this modelling approach and its biological validation are limited by the information about diseases, drugs, and the data available in public repositories. Whilst much is known about the signalling pathways affected by dexamethasone and tocilizumab, there is much unknown about the disease mechanisms for COVID-19. Our study was performed in July 2020, only 4 months into the pandemic; thus, we could have omitted updated information associated to new viral variants of concern, host variability, further understanding or better descriptions of SARS-CoV2 ARDS pathophysiology or high-throughput data-based datasets, that could have influenced the predicted results. In this sense, a recent review by Osuchowski et al. [85] summarizes the current knowledge on COVID-19 pathophysiology and phenotypes, highlighting the importance of immune-inflammatory processes, along with endothelial changes that mediate pulmonary disease, as well as other complications. Despite our data compilation did not categorize endothelial pathology as a separated item and our models were not build specifically focusing on endothelial or coagulation processes, our results highlight the involvement of several endothelial and coagulation mediators in the mechanisms of action of the drugs, specifically found to be modulated by dexamethasone (SELE, SELP) and tocilizumab (ACE2, AGT1R, ANGPT2, CDH5, EGFR, FN1, TGFA and VEGFA). As commented, the study by Ullah et al. [67], highlights the role of tocilizumab in improving performance on an institutional treatment including dexamethasone over coagulopathy control, supporting our model results. Furthermore, the evidence regarding the use of biologics targeting IL-6R –including tocilizumab–and IL-6 downstream JAK proteins since we started this study has increased [40, 41, 86–90], to the point that its use is recommended in the NIH guidelines for treatment of hospitalized patients requiring oxygen or assisted ventilation or oxygenation, in combination to dexamethasone [91] The methodology employed for the modelling, considering the whole human protein network and a wide range of drug-pathology relationships for the training (S4 Table), allow the TPMS-based models to infer biological and functional information on not-so-well documented therapeutic areas by connecting a wider biological knowledge spectrum, as shown in previous studies [47, 55, 83, 84].Therefore, while the models and conclusions could be updated over time as new information is generated, potentially improving accuracy of the results and allowing exploring unanswered questions in COVID-19 evoked ARDS, our approach has provided results that are in agreement with current molecular pathophysiological and clinical knowledge, providing protein candidates as response biomarkers and supporting the role of the approach in exploring mechanistic insights on the effects of current available therapies.

## 5. Conclusions

Our *in silico* modelling results showed that the corticosteroid dexamethasone has a wide and pleiotropic immunomodulatory and anti-inflammatory effect in COVID-19 evoked ARDS, supporting its use to ensure an effective control of the cytokine storm, as well as other ARDS signs and processes such as pneumonia and fibrosis. IL-6 receptor blockers, such as tocilizumab, have additional effects on the hyperinflammatory state leading to COVID-19 evoked ARDS. Our models support and provide mechanistic insight into the synergistic effect of combined treatment of severe patients with dexamethasone and IL-6 blockers to achieve a strong immune-modulatory effect that may attenuate hyperinflammation and disease worsening, mainly mediated by the JAK/STAT and NF-κB pathways. Additional data on COVID-19 pathophysiology will allow to fine tune AI-bases approaches as the one presented here.

## Supporting information

S1 Table Summary of the motives identified as involved in COVID-19 evoked ARDS. The amount of protein effectors identified in each motive are noted.
(XLSX)

S2 Table Proteins included in the COVID-19 evoked ARDS molecular characterization. Activation in COVID-19 evoked ARDS column indicates whether the protein is increased/overactivated (1) or reduced/inhibited (-1) in the context of COVID-19. Note that proteins can be repeated in more than one motive.
(XLSX)

S3 Table Summary of the drug characterization at bioflags level. Dexamethasone and tocilizumab bioflags considered in this project. The “Effect” column indicates whether the drug induced an inhibition (red arrow) or activation (green arrow) of the said bioflag.
(XLSX)

S4 Table TPMS training set. Summary of data (number of entries in the database for each data type) used for model construction (network and training set).
(XLSX)

S5 Table Summary of the sources of information found in the scientific literature supporting the predicted mechanisms for dexamethasone. Activation induced by Node A to Node B column indicates whether the first node of the interaction (Node A) activates (1) or inhibits (-1) the activity of the second node of the interaction (Node B).
(XLSX)

S6 Table Summary of the sources of information found in the scientific literature supporting the predicted mechanisms for tocilizumab. Activation induced by Node A to Node B column indicates whether the first node of the interaction (Node A) activates (1) or inhibits (-1) the activity of the second node of the interaction (Node B).
(XLSX)

S7 Table Complete enrichment results. for the predicted model of tocilizumab and dexamethasone in COVID-19evoked ARDS.
(XLSX)

S1 File Cytoscape session. Cystoscape session displaying COVID-19 evoked ARDS interactome (direct links from COVID-19 evoked ARDS effectors), and subnetworks centred around dexamethasone or tocilizumab targets.
(CYS)

## Abbreviations

ARDS: acute respiratory distress syndrome
COVID-19: coronavirus disease 2019
GR: glucocorticoid receptor
IL-6: interleukin 6
IL6RA: interleukin 6 receptor
MoA: mechanism of action
RCT: randomized clinical trial
SARS-CoV-2: severe acute respiratory syndrome coronavirus 2
TPMS: Therapeutic Performance Mapping System
